# Health promotion challenges and opportunities in the Western Balkans: a review of contemporary policies and actions

**DOI:** 10.3389/fpubh.2025.1666353

**Published:** 2026-01-27

**Authors:** Jonila Gabrani, Alexander Rommel, Aline Anton, Šeila Cilović-Lagarija, Dragana Grujić-Vujmilović, Martin Petrovski, Natasa Rosic, Natasa Terzic, Sinisa Skočibušić, Milena Šantrić-Milićević

**Affiliations:** 1 Center for Scientific Research in Public Health, Faculty of Medical Technical Sciences, European University of Tirana, Tirana, Albania; 2 Department of Epidemiology and Health Monitoring, Robert Koch Institute, Berlin, Germany; 3 Institute for Public Health of the Federation of Bosnia and Herzegovina, Sarajevo, Bosnia and Herzegovina; 4 Department of Social Medicine, Public Health Institute of the Republic of Srpska, Banja Luka, Bosnia and Herzegovina; 5 Department of Social Medicine, Faculty of Medicine, University of Banja Luka, Banja Luka, Bosnia and Herzegovina; 6 Institute of Public Health, Skopje, North Macedonia; 7 City Institute of Public Health Belgrade, Belgrade, Serbia; 8 Institute of Public Health of Montenegro, Podgorica, Montenegro; 9 Institute of Social Medicine, Faculty of Medicine, University of Belgrade, Belgrade, Serbia; 10 Laboratory for Strengthening the Capacity and Performance of Health Systems and Workforce for Health Equity, Faculty of Medicine, University of Belgrade, Belgrade, Serbia

**Keywords:** community engagement, health promotion, Ottawa Charter, preventive care, public health policy, Western Balkans

## Abstract

**Background:**

Countries in the Western Balkans (WBCs) fall behind the European Union in implementing effective health promotion. This study explores the key components of national health promotion policies and action plans in Albania, Federation of BiH, and the Republic of Srpska, Montenegro, North Macedonia, and Serbia, with the aim of describing key action trends, gaps, and challenges, and recommendations for health promotion implementation improvement.

**Methods:**

Within the framework of the “Western Balkan Strategic Partnership for Health Protection” (WASP) project, implemented in five WBCs on behalf of the Global Health Protection Programme (GHPP), 2023–2025, and using the” Ottawa Charter for Health Promotion” a descriptive observational study was employed, combining data and information provided from the desk review of national legal frameworks, country-specific reports and consultative meetings to describe health promotion policies and action plans implementation in the period from 2010 to 2022.

**Results:**

WBCs highlight diverse national priorities, such as promoting education and healthy lifestyles (e.g., Albania, Serbia, Federation of BiH, and the Republic of Srpska), addressing health inequalities (e.g., Federation of BiH, and the Republic of Srpska, Serbia), and digital health systems. The strategies and action plans of WBCs have common health promotion goals, and challenges. Community engagement and enforcement of public health policies are insufficient. Preventive care services are notably underdeveloped in rural areas with inadequate healthcare infrastructure. Although legal frameworks show that there is commitment to health promotion, putting action plans into practice is still a challenge, especially when it comes to involving communities and focusing on prevention. Budget transparency and working together across the region could also be improved to better tackle health inequalities and build stronger health systems.

**Conclusion:**

While WBCs demonstrate growing political commitment to health promotion, substantial gaps remain in translating strategies into sustainable action. Strengthening intersectoral collaboration, improving monitoring systems, securing stable financing, and expanding community participation are crucial for advancing equitable, prevention-oriented health systems across the region.

## 1 Introduction

### 1.1 Background and rationale

Over the past three decades, countries in the Western Balkans (WBCs) have undergone profound demographic, social, economic as well as epidemiological transformations. These shifts have created both challenges and opportunities for advancing health promotion as a core component of public health policy (1). In response to emerging health threats, the WBCs have increasingly prioritized health promotion in the frame of primary healthcare strengthening within their national agendas (2, 3). However, no comprehensive regional study has reviewed the legal frameworks, strategic objectives, and action plans for health promotion in the Western Balkan countries (WBCs), using the Ottawa Charter for Health Promotion as a conceptual and analytical framework. This represents a critical research gap, as understanding how the Charter’s principles are reflected in national policies is essential for assessing progress, identifying implementation gaps, and informing future regional and EU-aligned health promotion strategies (4).

Similar to other regions, the Western Balkans face a rising burden of non-communicable diseases (NCDs). Major conditions include cardiovascular diseases, cancer, diabetes, and chronic respiratory illnesses. These are largely caused by modifiable lifestyle factors—tobacco use, unhealthy diets, physical inactivity, and excessive alcohol consumption (5, 6). Health promotion and disease prevention efforts are therefore essential to reduce this burden, alongside quality-of-care improvement and primary healthcare service utilization (7). Health promotion not only addresses behavioral risk factors but also empowers individuals and communities to make informed decisions and adopt healthier lifestyles (4).

An additional dimension of this context is the shared aspiration of the WBCs to join the European Union (EU). This aspiration entails alignment with EU public health standards and regulatory frameworks. Key instruments include Directive 2003/33/EC on tobacco advertising (8), Regulation (EU) No 1169/2011 on food information for consumers (9), and action plans targeting obesity, alcohol use, and mental health (10, 11). The EU also provides strategic guidance on nutrition, health systems strengthening, and cross-border health information exchange (12, 13).

The Ottawa Charter for Health Promotion, introduced in 1986, marked a paradigm shift from biomedical to biopsychosocial approaches to public health, emphasizing empowerment, equity, and multisectoral action (4). The Charter’s five action areas structured our comparative analysis. The framework emphasizes key action areas that guide the assessment and analysis of health promotion efforts in the WBCs.

### 1.2 Evidence before this study and what it adds

Previous research on health promotion in the WBCs has been limited and largely fragmented, often focused on disease-specific interventions or individual risk factors rather than comprehensive, policy-level, and national strategies. Analyses of national health systems have also pointed to insufficient resource allocation, poor intersectoral coordination, and a lack of standardized indicators for monitoring health promotion efforts (13–15).

Comparative assessments show persistent gaps between EU member states and the Western Balkans in implementing EU-aligned public health policies. Although the EU has introduced several frameworks—such as the Action Plan on Childhood Obesity and the European Alcohol and Health Forum—data on their adaptation in the Western Balkan context remain limited (8–18). To date, no comprehensive regional study has reviewed the legal frameworks and strategic objectives and action plans for health promotion in the WBCs using the Ottawa Charter as a conceptual lens.

### 1.3 Aims and objectives

This study aims to describe the development of contemporary health promotion strategies and action plans, in the WBCs: Albania, Bosnia and Herzegovina (BiH), including the Federation of BiH and the Republic of Srpska—, Montenegro, North Macedonia and Serbia, to identify priorities, implementation challenges and the gap between legislative intent and practical outcomes.

Research questions:

(i) What are the main commonalities and differences across Western Balkan countries in terms of national legislation and health promotion strategies, action plans and to what extent do these documents articulate objectives, activities, budgets, indicators, and implementation mechanisms?
(ii) To what extent are health promotion actions in the WBCs aligned with the Ottawa Charter, and what challenges hinder their effective implementation?

## 2 Materials and methods

### 2.1 Study design and framework

This study employed a qualitative, cross-country comparative design to map and describe health promotion policy development in the WBCs over the last two decades, within the framework of the “Western Balkan Strategic Partnership for Health Protection” (WASP) project, which is implemented by the Robert Koch Institute on behalf of the German Federal Ministry of Health from 2023 to 2025 approach. This was an observational study that triangulated data and information sources (a purposive narrative review of the focused literature (desk research), a descriptive analysis of health promotion action plans, and a qualitative analysis of experts’ opinions).

A purposive narrative review was undertaken, grounded in a comprehensive literature search across PubMed/Medline, Scopus, and Google Scholar. This search encompassed original articles and reviews published in English and various domestic languages from 2019 to 2024. The literature search was conducted collaboratively by two authors from each participating country.

The search employed a range of keyword combinations that reflected specific criteria related to: the population (Western Balkans, Balkan countries, Southeast Europe, and specific nations), context (public health policies, strategies, action plans, and objectives), and purpose (health promotion and improvement, principles of the Ottawa Charter, disease prevention, preventive care, and community engagement).

The selection process for included papers was based on comprehensive criteria, ensuring that only those addressing all three dimensions were retained, with authors JG and AR overseeing this process. Subsequently, the study utilized a legal mapping methodology, which is widely recognized in public health law research (16).

All authors engaged in a meticulous qualitative thematic and content analysis, adhering to a structured three-step approach. Step (i): Regular Consultative Meetings. WASP project partners met regularly to coordinate data collection, validate findings, and exchange institutional experiences.

Step (ii): Desk Review. A structured desk review assessed national legal frameworks and health promotion action plans. Each country’s documents were reviewed to evaluate their alignment with the Ottawa Charter (4). Documents were examined in native languages or translated into English. Priority was given to European WHO-endorsed action plans or those adopted by national governments (17–19). Key components reviewed included: the epidemiological and social context addressed in each plan; alignment between strategic objectives, indicators, and funding; and integration of equity-focused measures.

Step (iii): Consolidated template. Findings were synthesized into a shared consolidated template summarizing common trends and country-specific insights (Tables 1, 2). All analysis was performed manually. Each national focal point conducted preliminary data extraction and thematic coding for their respective country. The results were then compared and validated through joint review sessions involving all partners. The final synthesis and interpretation were coordinated by the lead author, the methodological co-lead, and the project leader, who ensured coherence, analytical rigor, and alignment with the Ottawa Charter framework.

**Table 1:** Regulatory framework: legal framework and main national strategies for health promotion and prevention in the Western Balkan countries.

ALB	BIH Federation	BIH Srpska	MKD	MNE	SRB
Law no. 10138, dated 11.05.2009 On public health.^1^	Law on Health Care (“Official Gazette of the Federation of Bosnia and Herzegovina,” no. 46/10 and 75/13).	Law on Health Care Off Gazzete No 57/22	Law on Health Care Off. Gazette No 43/2012 with last amendment Off. Gazette No 37/2016	Law on Health Care (Off. Gazette of MNE No 003/23)	Law on Health Care: 25/2019–40, 92/2023–384
Decision of Council of Ministers no. 101 dated 4.2.2015. Health Services Package, in the public PHC funded by the Compulsory Health Care Insurance Fund.^2^	Law on Health Insurance of the Federation of Bosnia and Herzegovina (“Official Gazette of the Federation of Bosnia and Herzegovina,” no. 30/1997, 7/2002, 70/2008, 48/2011, 100/2014 - decision US, 36/2018 and 61/2022),	Law on obligatory health insurance Off Gazzete No 93/22	Law on Public Health Off. Gazette No 22/2010 with last amendment Off. Gazette No 37/2016	Law on mandatory Health Insurance (Off. Gazette of MNE No 145/21)	Law on Public Health: “Official Gazette of RS,” number 15 of February 25, 2016.
Law no. 8652, dated 31.7.2000, On organization and functioning of the local government” (as amended by law no. 9208, dated 18.3.2004).	Law on Health Records Official (Gazette of the Federation of Bosnia and Herzegovina no. 37/2012)		Law on Health Statistics Off. Gazette No 20/2009 with last amendment Off. Gazette No 150/2015	Law on health data collections (Off. Gazette of MNE No 40/11)	Law on Health Insurance Official Gazette of the RS,” no. 25 of April 3, 2019, 92 of October 27, 2023
Decision of Council of Ministers No. 419, dated 4.7.2018, On the Establishment, Organization Functioning of the Health Care Services Operator			Law for Mental Health Off. Gazette No 71/2006, with last amendment Off. Gazette No 150/2015	Regulations on basic benefit package within mandatory health insurance (Off. Gaz. No 103/20)	Law on health documentation and records in the field of health: 92/2023–254
			Law on opioid drugs and psychotropic substances Off. Gazette No 103/2008 with last amendment Off. Gazette No 37/2016	Decision on health care network (Off. Gaz. No 049/23)	Law on Official Statistics: (“Official Gazette of RS,” No. 104/2009)
National Health Strategy 2016–2020	Strategy of the Federation of Bosnia and Hercegovina 2021–2027.	Action Plan for prevention and control of non-communicable diseases in the Republic of Srpska for the period of 2019–2026.	National Health Strategy 2021–2030	Strategy for health system development 2023–2027 with Action plan 2023/24	Public health strategy in the Republic of Serbia 2018–2026. year: 61/2018–6
National NCD control plan 2016–2020,	Action Plan for the Prevention and Control of Chronic Non-Communicable Diseases in the Federation of Bosnia and Herzegovina for the period 2019–2025.		National Strategy for Mental Health 2018–2025	Program for control and prevention of NCDs in MNE, 2019–2021	Strategy for prevention and control of chronic non-communicable disease in the Republic of Serbia: 22/2009–46
Health Promotion Action Plan 2017–2021	Strategy for the Advancement of the Rights and Status of Persons with Disabilities in FBiH 2016–2021 https://fmoh.gov.ba/stranica/21/strategije-i-politike		National Strategy for Drugs 2014–2020, National Strategy for Drugs 2021–2025 with Action plan until 2023	Strategy for improvement of integral health information system and e-health in MNE, 2018–2023	Action plan for the period from 2018 to 2026 for implementing the Public Health Strategy. Public Health Strategy in the Republic of Serbia 2018–2026. “Official Gazette of RS,” no. 61/2018.
Action Plan on NCDs, Albania 2021–2030	Resolution on Diabetes (The House of Peoples of the Parliament of the Federation of Bosnia and Herzegovina, at its 11th session held on November 15, 2012, adopted the Resolution on Diabetes.) https://fmoh.gov.ba/stranica/21/strategije-i-politike		National Strategy for NCD, 2009	National Strategy for Sustainable Development, 2017–2030	Mental health protection program in the Republic of Serbia for the period 2019–2026. year: 84/2019-201Official Gazette of the RS,” No. 84 of November 29, 2019.
	Strategy on Rare Diseases in FBiH 2014–2020 (Upon the proposal of the Federal Ministry of Health, the Government of FBiH, at its 120th session held on June 19, 2014, gave consent to the Strategy on Rare Diseases of the Federation of Bosnia and Herzegovina (2014–2020), which was developed with the support of the European Commission Office for BiH.) Strategic Plan for the Improvement of Early Growth and Development of Children in FBiH 2013–2017 Policy for Improving Children’s Nutrition in FBiH Policy and Strategy for the Protection and Improvement of Mental Health in the Federation of Bosnia and Herzegovina (2012–2020) Strategy for the Prevention, Treatment, and Control of Malignant Neoplasms in FBiH 2012–2020 Strategy for the Improvement of Sexual and Reproductive Health and Rights in FBiH		National Strategy for Health Informatics System until 2020, 2007		Digitization program in the healthcare system of the Republic of Serbia for the period 2022–2026 Based on Article 38, Paragraph 1 of the Law on the Planning System of the Republic of Serbia (“Official Gazette of the RS,” No. 30/18),

Source: Authors compilation.

^1^Document view - Center for Official Publications.

^2^VKM_NR_101_DATE_04_02_2015_PAKETA_E_PARESORIT.pdf.

**Table 2:** Health promotion strategies and disease preventions in Western Balkans (main objectives, activities & indicators).

Dimensions	ALB	BIH Federation	BIH Srpska	MKD	MNE	SRB
The vision of the Health Promotion Action	Vision: Healthy life and well-being for the entire Albanian population and all the other actors.	Strategy of the Federation of Bosnia and Hercegovina 2021–2027. (And within it is presented the part related to Empowering the potential of preventive medicine.)	Promotion of healthy consumption through fiscal and marketing policies	In 2017, a national action plan aligned with the EU’s NCD prevention strategy was drafted but never adopted. However, an earlier strategy on NCDs was approved in 2009, aiming to reduce premature deaths, lessen the NCD burden, and improve both quality of life and health equity across population groups.	Effective health promotion and disease prevention through control of risk factors, improvement of multisectoral collaboration and engagement of the whole society	Vision: Protection and improvement the population health,reduced inequalities in health and joint actions of government and society for health and well-being
Strategic objective	OB1. 1: Increasing the health awareness of the Albanian population and orientation toward a healthy lifestyle and proper use of health services. OB2. Strengthening supportive environments and promoting efficient interventions for the implementation of education and health promotion programs. OB3. Creating resilient communities for the protection and promotion of health and well-being.	Overview of strategic goals, priorities and measures 2.2. To improve outcomes of the health system 2.2.1. Improve access and to reduce inequalities in health services 2.2.2. Strengthen potential of preventive medicine 2.2.3. Strengthen informatization and digitalization of the health protection system 2.2.4. Enhance actions in public health crisis situations 2.2.5. Strengthen financial sustainability of the health system and to enhance fairness in financing health protection 2.2.6. Create an environment for scientific-research work and bio-medical research	Prepare and implement a comprehensive social marketing campaign for: promoting healthy consumption, reformulation and improvement of products (salt, fats and sugars); active life and mobility; clean air; oral health and health of the musculoskeletal system; mental health; health in specific settings. Preparation and Implementation of fiscal and marketing policies to influence on demand, access and affordability for tobacco, alcohol and foods and drinks high in saturated fats, trans fats, salt and sugar. Create an environment to enable and promote a healthy lifestyle	Specific goals include: 1. Prevent and/or delay the occurrence of NCDs; 2.reduce the development and complications of NCDs 3.improve the quality of life of patients, their families and the people who care for them 4.reduce hospital and inpatient admissions of adults 5.reduce inappropriate variations in medical practice	OB2: Health promotion – advocacy for health and community involvement in order to eliminate or reduce exposure to behavioral risk factors OB2.1. Awareness of the population about the importance of prevention and control of non-communicable diseases, and familiarization of the public with the activities carried out in within this area OB2.2. The most effective prevention of non-communicable diseases resulting from the elimination or reduction of population exposure to risk factors for NCDs: tobacco use, harmful use of alcohol, unhealthy diet and insufficient physical activity. OB2.3. The need for coordination of action plans for the prevention of the mentioned risk factors OB2.4. Strengthening individual information on healthy lifestyles, prevention, availability of health services, as well as involvement of local communities, for more comprehensive response;	Objective: 4.1. Improving health and reducing health inequalities Objective 4.2: improvement of the living and working environment Objective 4.3: Prevention and suppression of diseases from the leading health risks Objective 4.4 Development of health promotion actions in the community Specific objective: 4.4.1. Improving the knowledge and behavior of the population in relation to preserving and improving health and reducing risk factors 4.4.2. Improvement of partnership and social inclusion for health in the local community - application of the mechanism for integrated management 4.4.3. Developing and strengthening a network of environments that support health and healthy choices.
Measurable Indicators defined yes/no	Yes	No	No	No	Yes (defined but without final expected outcome)	Yes, defined
a. Narrative Budget b. Action Plan Matrix c. Costs	a. YES, activity-based costing method b. YES c. yes	a. Through the routine work (state budget), b. Yes c. N/a or not defined	a. Through the routine work b. Yes c. Not defined in document	a. Trough the routine work (state budget) b. No c. Not defined	a. Yes, trough the routine work (State Budget) and donations b. Yes c. Not defined	a. Yes, State Budget according to the activities predict in the Action Plan b. Yes c. Not defined

Source: Authors compilation.

The study used the Ottawa Charter for Health Promotion as a guiding analytical framework to describe national strategies, legal instruments, and health promotion action plans implementation mechanisms across five countries: Albania, Bosnia and Herzegovina (including the Federation of BiH and Republic of Srpska), Montenegro, North Macedonia, and Serbia.

### 2.2 Setting and timeframe

The study focused on five WBCs participating in the WASP project. Data collection and analysis were conducted between October 2023 and November 2024.

### 2.3 Study instruments and analytical framework

The Ottawa Charter for Health Promotion, introduced in 1986, was used as the conceptual framework (10). The Ottawa Charter focuses on enabling people to take control over and improve their health through the following action areas: (1) building healthy public policy, (2) creating supportive environments, (3) strengthening community action, (4) developing personal skills, and (5) reorienting health services (see Appendix 1 for explanations of each area/action).

Aligned with the five action areas of the Ottawa Charter, a structured review template was created to guide data collection across WASP participating countries, and synthesis regarding the Charter’s five actions (4).

### 2.4 Study units, variables, and data sources

The primary study units in the review were national regulatory frameworks, strategic documents, and action plans related to health promotion in the five countries. Key variables reviewed included (i) Legal and institutional frameworks for health promotion; (ii) Strategic objectives and foreseen activities; (iii) Budget allocation and funding mechanisms; (iv) Indicators for monitoring and evaluation; (v) Reported outcomes and implementation gaps. The selection of variables was grounded in the Ottawa Charter for Health Promotion and the WHO Health System Building Blocks framework (17). These frameworks provided the conceptual basis for assessing how countries translate political commitment into practical health promotion actions.

Each variable captured a distinct aspect of policy design and implementation. Examining legal and institutional frameworks helped assess political commitment and the extent of alignment with the Ottawa Charter actions. Reviewing strategic objectives and activities showed how national priorities are operationalized in practice. Analysis of budget allocations and funding mechanisms offered insight into financial commitment and the sustainability of interventions. Monitoring and evaluation indicators were used to explore systems of accountability and performance assessment, while reported outcomes and implementation gaps revealed barriers to translating policy into action and areas for improvement.

The desk search (in the national languages and English) included the following keyword combinations: health promotion, WBCs, Ottawa Charter, action plans, public health policy, non-communicable diseases, legal framework, and community health.

Data sources included published and unpublished national and international literature, national legal databases, health strategies, action plans, and WHO and EU policy documents (2, 3, 6, 14–18). Additional materials were retrieved via PubMed, Google Scholar, national ministry websites, and grey literature shared by WASP partners.

### 2.5 Ethical considerations

This study used secondary data from publicly available documents and institutional workshops under the WASP project. No human participants were involved. Ethical clearance was therefore not required, in line with the Declaration of Helsinki and journal guidelines (19).

## 3 Results

The results are presented in three parts, reflecting the analytical sequence outlined in the Methods section. Step I reviews the evolution of national legal frameworks; Step II summarizes strategic objectives, implementation mechanisms, and budgetary trends; and Step III compares the extent to which each country operationalized the five action areas of the Ottawa Charter for Health Promotion.

### 3.1 Regulatory frameworks and policy commitment

Across the WBCs, notable progress has been made in revising national legislation to align with European Union standards, particularly in the domain of public health.

Serbia was the first Western Balkan country to adopt a law on public health in 2008; Albania adopted a law on public healthcare in 2009, while North Macedonia enacted the law on public health in 2010. The new legislation was built to be in line with the EU directives and legislation. Bosnia and Herzegovina adopted in 2010 the roadmap for EU integration of the sector.

Table 1 assembles the main documents from our desk research, on Regulatory Frameworks and Main National Strategies for Health Promotion and Prevention in Albania, Federation of Bosnia and Herzegovina, Republic of Srpska, Montenegro, North Macedonia and Serbia. Table 1 shows that while all Western Balkan countries have adopted public health laws, the scope and enforcement mechanisms differ substantially across jurisdictions (see Appendix 1).

These legislative actions align partly with EU acquis requirements, especially in areas such as compulsory health insurance, health information systems, and essential service packages. However, convergence remains uneven due to political decentralization and limited institutional capacity. EU competences in health are limited to selected areas such as tobacco control and consumer safety; therefore, harmonization largely remains a voluntary process (20, 21).

#### 3.1.1 Strategic planning for health promotion and disease prevention

The WBCs show strong intent to advance public health through national strategies targeting health promotion and NCD prevention. Albania and Serbia have adopted multi-year strategies with clear implementation frameworks. Montenegro and North Macedonia have also made progress, though continuity gaps exist, as seen in North Macedonia’s expired strategy for prevention and control of non-communicable diseases.

#### 3.1.2 Digital health and information system reform

Digital health reform is a growing priority. Serbia, Montenegro, and North Macedonia have launched digitization strategies to support data-driven governance. Despite clear planning, challenges in implementation—such as infrastructure and resource limitations—highlight the need for sustained investment and continuity planning. Building on these legislative frameworks, the next section examines how strategic objectives were formulated, resourced, and monitored across the Western Balkans.

#### 3.1.3 Strategic planning and implementation

The analysis from Table 2 reveals a range of visions and strategies across the WBCs regarding health promotion. It highlights shared priorities such as lifestyle modification and health literacy, but also reveals large variations in measurable indicators and budget transparency (see Appendix 1).

Strategic objectives across the WBCs converge on key areas: improving population awareness, supporting healthy environments, and integrating community engagement (see Table 2). For example, Albania focuses on lifestyle awareness, programmatic interventions, and community resilience. North Macedonia prioritizes reducing inequalities and enabling partnerships at the community level. Serbia outlines structured objectives from health education to local network development.

Implementation activities include, among else, campaigns on healthy diets, physical activity, and air quality (Federation of BiH, Montenegro), fiscal and marketing policies to curb harmful consumption (Federation of BiH), and integration of promotion into primary health services (Montenegro, Serbia, Albania).

Budgetary transparency and implementation capacity, however, remain inconsistent. While Albania and Montenegro provide specific budget allocations—including Albania’s activity-based costing —most other countries either report funding through routine state budgets or do not define financial resources in their strategic documents. This variability limits the ability to evaluate resource adequacy and cost-effectiveness of health promotion interventions.

Monitoring and evaluation mechanisms also differ significantly. Albania, Montenegro, and North Macedonia incorporate measurable indicators in their action plans, though only Albania provides outcome-linked metrics. Bosnia and Herzegovina, and Serbia show less consistency in indicator use, limiting performance tracking. Furthermore, documentation of achieved outcomes is sparse, with most countries reporting only partially on implementation progress or leaving achievement status undefined.

Persistent challenges include limited monitoring and evaluation frameworks, a lack of standardized indicators across countries, and variations in budget clarity. Despite these challenges, there is a growing awareness of the importance of integrating health promotion into broader public health policy both politically and institutionally. Strengthening monitoring systems, enhancing intersectoral coordination, and securing sustainable financing are critical next steps to improve health promotion governance across the WBCs.

### 3.2 Actions alignment with the Ottawa Charter

Following the assessment of national strategies, step three explores how these actions correspond to the Ottawa Charter’s five domains (see Tables 3, 4).

**Table 3:** Country specific actions within Ottawa Charter for Health Promotion, emphasized by each country.

Ottawa charter action	Observed gaps/areas for strengthening	Suggested improvement measures
Building healthy public policy	Implementation of health policies remains uneven, partly due to limited enforcement of tobacco- and alcohol-control measures. Intersectoral collaboration between health and other policy areas is still developing.	Enhance mechanisms for policy enforcement and accountability. Integrate health-promotion goals across sectors. Allocate targeted resources for implementation, especially in underserved areas.
Creating supportive environments	Gaps in infrastructure and workforce availability, particularly in rural settings. Health-education programs are not yet systematically embedded.	Expand access to preventive and primary-care services in all communities. Institutionalize school- and community-based health-promotion programs.
Strengthening community action	Community participation in health initiatives remains limited. Local capacity for planning and sustaining health actions needs further support.	Encourage participatory community engagement and training. Involve local actors in health-policy development. Foster partnerships between civil society and the public sector.
Developing personal skills	Health literacy and awareness of NCDs, sexual, and mental health are still suboptimal Many health-promotion activities are short-term or project-based.	Implement sustained, age-appropriate health-education initiatives. Use digital and community-based tools to strengthen self-care and preventive behavior.
Reorienting health services	Services remain oriented mainly toward curative rather than preventive care. Preventive services are not yet fully integrated into primary health-care delivery.	Strengthen the preventive focus of primary care. Integrate screenings, vaccinations, and counseling into routine service packages. Expand training in preventive and population-health approaches.

Source: Authors compilation.

**Table 4:** Countries’ alignment with the five Ottawa Charter action areas.

Country/Entity	Examples of strong alignment	Examples of weak alignment	Illustrative/Notes
Albania (ALB)	Building Healthy Public Policy – comprehensive legislation on public health, NCD prevention, and tobacco control; multi-sectoral action plans adopted.	Creating Supportive Environments – Efforts to enhance access to health-promoting settings—such as safe (green) public spaces and healthy food environments—remain a key area for development.	Strong legal and policy base, but environmental health promotion remains underdeveloped.
Federation of Bosnia and Herzegovina (FBiH)	Building Healthy Public Policy – tobacco-control legislation. Strengthening Community Action – local health-education initiatives.	Creating Supportive Environments – limited healthy food options and lack of air-purification systems in schools.	Solid policy on tobacco-control legislation and comprehensive legislation on public health issues (NCD and CD disease) but limited environmental implementation.
Republic of Srpska (RS)	Building Healthy Public Policy – tobacco-control laws. Creating Supportive Environments – community-based initiatives promoting healthy lifestyles.	Developing Personal Skills – absence of long-term health-education programmes and materials.	Strong policy intent; limited continuity in capacity-building.
Montenegro (MNE)	Building Healthy Public Policy – updated National Health Programme and tobacco-control measures.	Reorienting Health Services – fragmented delivery and limited integration between preventive and curative services.	Policy progress is not yet mirrored by systemic coordination.
Serbia (SRB)	Building Healthy Public Policy – comprehensive legislation, programmes, and budgets. Strengthening Community Action – NGO and community-based funding mechanisms.	Reorienting Health Services – insufficient integration and support for informal caregivers.	Strong governance; requires structural service reform.
North Macedonia (NMK)	Building Healthy Public Policy- Laws regulate food safety, occupational health, air quality, and vaccination programs.	Creating Supportive Environments -improvements in environmental protection, pollution control, and urban planning, but air quality (especially in Skopje and Tetovo) remains a major issue.	Intersectoral collaboration exists through the Ministry of Health, Ministry of Education, and Ministry of Environment, though enforcement and coordination sometimes lag.

Table 4 summarizes self-assessment of countries alignment with the five Ottawa Charter action areas. Countries align strongest in developing legislation and strategies—particularly in Building Healthy Public Policy—while persistent weaknesses are found in Creating Supportive Environments, Reorienting Health Services, and Developing Personal Skills.

As shown in Table 4, most Western Balkan countries demonstrate substantial progress in establishing legislative and strategic frameworks for health promotion. Albania, Serbia, the Federation of BiH, and Montenegro stand out for their strong public policy foundations, reflecting advanced tobacco-control laws, NCD prevention plans, and multi-sectoral coordination mechanisms. In contrast, Creating Supportive Environments and Reorienting Health Services emerge as common weaknesses across the region. These shortcomings stem largely from insufficient infrastructure, limited intersectoral investment, and the lack of mechanisms connecting health, education, and environmental sectors. Overall, the findings underscore a pattern of *policy maturity but implementation fragility* across all Ottawa Charter pillars.

#### 3.2.1 Creating supportive environments

Montenegro, North Macedonia, and Albania have worked to promote environments that support healthy behaviors, particularly through school-based initiatives and local health promotion campaigns. Despite these efforts, challenges persist in rural areas, where healthcare infrastructure and workforce capacity are limited. Addressing these disparities requires targeted investments to improve service access and the expansion of structured health education programs at the community and school levels.

#### 3.2.2 Strengthening community action

Albania and North Macedonia have started engaging communities in health promotion, although progress remains uneven. A lack of sustained community involvement and limited local capacity to organize health initiatives hinder broader participation. Moving forward, building community leadership, providing training, and fostering inclusive health policy dialogues could enhance local ownership and improve outreach to underserved populations.

#### 3.2.3 Developing personal skills

Albania, Serbia, Federation of BiH and North Macedonia have emphasized health education to build personal skills, particularly regarding lifestyle-related risk factors. However, current efforts are often fragmented and short-lived. There is also a noted gap in health literacy in rural and marginalized communities, especially in the Republic of Srpska and the Federation of BiH. To address these challenges, countries should invest in long-term, age-inclusive health educational programs and explore digital platforms to extend access to credible, user-friendly health information.

#### 3.2.4 Reorienting health services

Reform toward preventive care has gained traction in Albania, North Macedonia, Serbia, and the Federation of BiH, yet curative services still dominate primary care systems. Preventive services—such as routine screenings and vaccinations—are not fully integrated or prioritized. To shift this paradigm, health systems must reconfigure their service delivery models to embed prevention within primary care, enhance early detection mechanisms, and upgrade the training of healthcare providers in community-based, preventive approaches. While there is a presence of public health professionals in the Western Balkan region, there is a significant opportunity for enhancing their capacity through targeted development initiatives (22–24).

## 4 Discussion

This study aimed to identify and compare the policy and implementation landscape of health promotion in the Western Balkans, using the Ottawa Charter as an analytical framework. The results provide new evidence on how countries translate policy commitments into practice under differing institutional and socioeconomic conditions. Several studies have highlighted persistent health inequalities across the region, especially in rural and underserved communities, where health promotion services remain underdeveloped and access to preventive care is limited (1, 25). This study makes a significant contribution to the public health policy literature by offering the first comprehensive overview of health promotion frameworks in the WBCs through the lens of the Ottawa Charter. Later frameworks—such as the Bangkok Charter (2005) and the Shanghai Declaration (2016)—expanded global health governance concepts. Yet, both reaffirmed the Ottawa Charter’s foundational importance in guiding local and national public health policy (26, 27). Key contributions include identifying shared policy priorities (e.g., improving health literacy, addressing risk behaviors, and promoting digital health transformation), highlighting systemic implementation challenges (such as weak enforcement and limited rural outreach), and emphasizing the gap between legislative intent and practical outcomes. The study also provides region-specific recommendations for improving coordination, financing, and community engagement aligned with the EU standards.

While existing research has noted disparities in health outcomes and system capacities within the WBCs, our analysis specifically reveals the gap between formal legislative alignment and the actual implementation of EU-aligned public health policies. These legislative actions align partially with EU acquis requirements, although convergence remains uneven due to decentralization and limited institutional capacity. EU competences in health are limited to specific domains and rely largely on voluntary coordination rather than binding harmonization (20, 21).

While many countries demonstrate legislative and strategic intent, the practical implementation of health promotion actions remains fragmented, particularly in rural and underserved areas (1, 2, 13). Strategic objectives across the WBCs focus on raising awareness, creating supportive environments, and enhancing community participation. Implementation activities include awareness campaigns, regulatory measures, and integration of health promotion into primary care. Despite the recognition of health promotion’s significance, there are challenges in the effective implementation of action plans. These challenges include limited knowledge about the specifics of action plans, such as activities matrices, indicator measurement, and overall achievements and challenges. Overall, the desk review revealed a need for improved documentation and reporting on health promotion activities, particularly regarding the implementation of action plans. Clear reporting mechanisms and standardized indicators are essential for tracking progress and identifying areas for improvement.

A key strength across the region lies in the adoption of public health legislation and the development of national strategies aimed at reducing the burden of non-communicable diseases. Countries such as Albania, Serbia, Federation of BiH and Montenegro have demonstrated policy commitment by adopting laws and multi-year strategies that incorporate prevention and education (1–3, 13). However, implementation challenges persist, including weak enforcement of tobacco and alcohol control, and limited cross-sectoral integration. These findings align with earlier research that identified significant gaps between formal health policies and practical delivery, especially at the sub-national level (9, 15, 28, 29).

The study also shows moderate progress in the development of supportive environments for health. Initiatives in Montenegro, North Macedonia, and Albania have focused on school-based health education and promoting healthy lifestyle. Yet, the limited reach in rural areas and insufficient infrastructure underline the need for equitable access. Similar challenges were noted in previous assessments across Eastern Europe, where supportive environments were hindered by resource constraints and uneven municipal capacities (1, 29, 30). Ensuring local investment and reinforcing community-based health systems are vital to strengthening this Ottawa Charter action area.

Community engagement, a cornerstone of sustainable health promotion, remains underutilized across the WBCs. This reflects wider trends seen in other Low- and Middle-Income Countries (LMIC) contexts, where community action is often deprioritized in favor of centralized, top-down interventions (10, 15). Capacity-building for local actors and institutional support for participatory health governance should be prioritized. Greater inclusion of civil society and municipal authorities in the design and implementation of health promotion efforts can foster both accountability and contextual responsiveness (1, 3, 25, 30).

Similarly, efforts to build personal skills through health education campaigns exist but are often fragmented and short-term. While Albania, Serbia, and North Macedonia have initiated programs targeting behavior change, these interventions frequently lack long-term sustainability or structured health literacy components. This is consistent with prior findings that highlighted low health literacy levels in rural and marginalized areas of the region (1, 3, 25). Bridging the urban–rural divide in access to health information and leveraging digital tools for education may offer cost-effective pathways to improvement.

The reorientation of health services toward preventive care is underway in North Macedonia, Serbia, and the Federation of BiH and Albania. However, this shift remains in its early stages, with curative services continuing to dominate. Institutional inertia, limited primary care funding, and insufficient training of health professionals are critical barriers (1–3, 9). These barriers have been reported in comparative studies across post-socialist countries, where structural transition toward preventive healthcare remains incomplete (1, 25, 28, 30). Fully embedding preventive services into routine care, supported by policy incentives and health workforce development, will be essential for sustainable change.

Overall, while the Ottawa Charter continues to serve as a relevant and actionable framework, the study indicates a need for renewed political commitment, clearer implementation pathways, and stronger intersectoral collaboration. Addressing the persistent gaps in monitoring, budgeting, and community engagement will be essential for advancing equitable health promotion across the WBCs. Future research and practice should prioritize regional peer learning, transparent evaluation frameworks, and the co-design of health interventions with affected communities.

In addition, in the Western Balkans, the coordination among various stakeholders in health promotion can occasionally be loose and non-transparent. This situation often results in a dispersion of responsibilities, which may hinder the effectiveness of overall health promotion initiatives. Effective coordination among stakeholders in health promotion is essential, yet it can sometimes be less cohesive and transparent than desired. This occasional dispersion of responsibilities may present challenges that impact the overall effectiveness of health promotion initiatives. Fostered communication and collaboration can significantly enhance our collective efforts in this important area.

To address these challenges, stakeholders should adopt proactive, evidence-based approaches. WHO guidance highlights the need for population-based interventions and actions addressing social determinants of health (29). Western Balkan countries could introduce social cards, as outlined in Serbia’s Law on Social Cards. Although Serbia’s law took effect in 2022, implementation remains pending. Once operational, it would enable fairer access to social rights, especially in health promotion and disease prevention. However, the lack of clear governance, evaluation mechanisms, and transparent funding continues to hinder progress. Moreover, it is essential to implement structured health promotion frameworks such as the Tannahill model (31) and the Albanian Health Promotion Model (32), which offer practical pathways to integrate policy, prevention, and community empowerment. Health promotion remains fragmented—“a poor baby without parents/guardians”—and sustainability is further challenged by emerging paradigms like Planetary Health and One Health, where funding streams are often opaque and difficult to trace.

The EU’s Health Promotion Knowledge Gateway provides updated knowledge, datasets, visualizations, and other tools to support national health promotion strategies (33). An example of its use in evidence-informed policymaking is Finland’s experience (34). Using EU-developed metrics can also help assess and improve the monitoring of WBC stakeholders’ performance in health promotion compared to the EU region (18).

The sociopolitical context of the Western Balkans—marked by post-conflict transitions, decentralization, and economic disparities—has significantly complicated the implementation of health promotion (28). Countries with fragmented governance structures and limited fiscal space faced competing priorities and interests. The migration of health professionals further erodes public health system capacity, implying that investments in disease diagnostics and treatment constrain health risks prevention and health promotion activities (22–24, 35).

Geopolitically, EU accession remains a major driver of reform in the Western Balkan region. Alignment with *Chapter 28 (Consumer and Health Protection)* has advanced legislative progress in areas such as tobacco control and food safety, yet the Western Balkan countries still lack stable financing and institutional mechanisms to sustain EU public health initiatives (36). Unlike EU members, studied countries mostly rely on donor-supported or project-based interventions, and less on domestic capacities to operationalize prevention and intersectoral approaches for health promotion coordination. Compared to the *EU Health Strategy 2021–2027* and *Europe’s Beating Cancer Plan*, the Western Balkans exhibit strong policy convergence but limited transparency in operationalization. EU programmes emphasize the need to integrate governance, have transparency in the actions and measurable accountability—elements that remain weak across the region, given that key institutions lack official reports of the health promotion plans implementation and funding, no external objective evaluation of their activities exists, and are publicly available (except one policy brief). Bridging these divides requires governance will, stakeholder capacity, and sustained investment in intersectoral approaches (37, 38). The small, community-driven models are the sparks that link health promotion with social cohesion and offer promising prospects for achieving the EU integration goals.

The studied countries do share best practices. Several promising practices demonstrate growing regional capacity in health promotion. In Serbia, community-based non-communicable disease (NCD) prevention initiatives—supported through national primary-care reforms and the World Bank-funded Health Efficiency Project—have expanded outreach to local populations and strengthened preventive services within family medicine networks (39). Montenegro has advanced tobacco-control reforms, implementing excise-tax adjustments and population-based monitoring of smoking behavior in alignment with WHO Framework Convention on Tobacco Control (FCTC) guidance (40). In Albania, innovative digital health-promotion campaigns targeting youth and children have successfully integrated mobile applications and school-based education to encourage healthy behaviors (41). With the aim of improving child health and nutrition and promotion of healthy behaviors among children the Federation has a number of preventive programmes as well, eg. preventive eye-screening and dental health examinations (42). Moreover, organized screening programmes — including structured population-based recruitment, cross-sector coordination, and community outreach — demonstrate how targeted, well-governed prevention initiatives can significantly improve early detection and equity in access across the Western Balkans (43, 44).

### 4.1 Study limitations

This study has several methodological limitations. First, the analysis relied exclusively on publicly available national documents and secondary data sources, which may not capture the most recent policy updates, subnational initiatives, or implementation practices. Variations in document quality, completeness, and availability across countries may have resulted in uneven levels of detail and comparability. Second, because no standardized monitoring or evaluation reports were available, the study could not assess the effectiveness of health promotion strategies or measure implementation outcomes. Third, the absence of harmonized indicators across countries limited the ability to conduct systematic comparisons aligned with the Ottawa Charter action areas. Fourth, the document review template, while structured, was not externally validated and may be subject to interpretive bias, despite joint review and cross-validation by national focal points. Finally, the study adopted a formative, descriptive approach and did not include primary data collection; therefore, findings reflect policy intent rather than operational performance. Future research incorporating mixed methods, stakeholder interviews, and validated assessment tools would provide deeper insights into implementation processes and impact.

## 5 Conclusion

Our study highlights that, despite substantial legislative and strategic efforts in the WBCs to advance health promotion, the translation of policy into practice remains uneven. These findings reinforce the broader pattern observed throughout the region: strong policy frameworks but uneven translation into practice. The Ottawa Charter provides a robust framework for understanding and evaluating these efforts, yet its full potential is not consistently realized across the region. Challenges in enforcement, monitoring, community participation, and funding continue to limit the impact of health promotion interventions.

Despite these challenges, the growing stakeholders’ commitment to national strategies and the gradual reorientation of health services toward prevention are encouraging signs. Countries such as Albania and Serbia have begun to institutionalize multisectoral approaches, and examples of emerging community-based initiatives in North Macedonia point to the value of local engagement. To build on these strengths, future efforts must prioritize capacity building at the local level, improved monitoring and evaluation systems, and regional collaboration to harmonize practices and share learning.

Strategic investment in health literacy, preventive services, and inclusive policymaking will be key to ensuring that health promotion contributes effectively to reducing non-communicable disease burdens and achieving sustainable public health outcomes across the region. Ultimately, health promotion in the WBCs must move from policy rhetoric to tangible, inclusive, and evidence-informed action to meet the evolving needs of its populations and align more closely with EU health and equity standards.

### 5.1 Recommendations for further research

Building on the observed gaps and variations in health promotion strategies across the WBCs, several avenues for future research are warranted. First, in the absence of official evaluation reports, conducting qualitative interviews with key stakeholders—such as health policymakers, public health practitioners, and civil society actors—can offer valuable insights into the context-specific barriers and successes and inform strategies for institutional capacity-buildings in health promotion in WBCs.

Secondly, there is a strong rationale for developing a regional monitoring framework that employs a standardized set of indicators to track progress in major disease prevention. A shared digital dashboard, accessible to both national and regional actors, could enhance performance transparency, support further development of evidence-based health promotion policies, and facilitate cross-country best practices exchange and collaboration.

Thirdly, strengthening national public health capacities for health promotion should prioritize enhancing the health and care workforce policies, including their recruitment and retention in adequate numbers, improving competencies and salaries, and providing safe, better working conditions, along with opportunities for career development and growth in and equitable ways. On the other side there is need to introduce behavior change mechanisms that orients the patients toward PHC and preventive service utilization, rather than hospital ones (45–47).

Finally, promoting policy harmonization and mutual learning across the region is crucial for attracting top international and global actors in health promotion. Developing open platforms for cross-country exchange—especially on budgeting, community-based program delivery, and evaluation—can encourage convergence toward implementing more effective and equitable public health policies. These efforts would not only improve national outcomes but also strengthen regional coherence and readiness for future EU integration.

## Data Availability

The original contributions presented in the study are included in the article/Supplementary material, further inquiries can be directed to the corresponding author.
