# Supplementary material for: Health promotion challenges and opportunities in the Western Balkans: a review of contemporary policies and actions
Source: Front Public Health. 2026 Jan 27;13:1666353. doi: 10.3389/fpubh.2025.1666353 (PMC12886418; doi:10.3389/fpubh.2025.1666353)
Supplement: Supplementary file 1 [file Supplementary_file_1.docx]

**Appendix 1. Ottawa Charter for Health Promotion Framework**

1. **Building Healthy Public Policy**:
   - Examine the development of health promotion policies in the WBCs, including laws, regulations, and strategies related to for example tobacco control, alcohol regulation, NCD prevention, and overall public health.
2. **Creating Supportive Environments**:
   - Assess how public health initiatives create supportive environments for healthy lifestyles, such as improving access to primary care, fostering healthier community settings, and enhancing the physical environment for health promotion.
3. **Strengthening Community Action**:
   - Evaluate the role of community involvement and local governance in implementing health promotion activities. In the WBCs, there are concerns about local-level resource limitations; this framework highlights how communities are engaged in health promotion programs.
4. **Developing Personal Skills**:
   - Focus on health education campaigns and initiatives that build health literacy, encouraging individuals to make informed decisions regarding their health. This aligns with efforts in the WBCs to address lifestyle risk factors like smoking, unhealthy diets, and physical inactivity.
5. **Reorienting Health Services**:
   - Analyze how healthcare systems in the region are shifting from treatment-oriented models to preventive care, focusing on early detection, screenings, and community health services.
